# The Utility of Post-Void Residual Volume versus Sphincter Electromyography to Distinguish between Multiple System Atrophy and Parkinson’s Disease

**DOI:** 10.1371/journal.pone.0169405

**Published:** 2017-01-06

**Authors:** Tatsuya Yamamoto, Masato Asahina, Yoshitaka Yamanaka, Tomoyuki Uchiyama, Shigeki Hirano, Miki Fuse, Yasuko Koga, Ryuji Sakakibara, Satoshi Kuwabara

**Affiliations:** 1 Department of Neurology, Chiba University Graduate School of Medicine, Chiba, Japan; 2 Department of Neurology, Continence Center, Dokkyo Medical University, Tochigi, Japan; 3 Department of Urology, Chiba University Graduate School of Medicine, Chiba, Japan; 4 Department of Molecular Diagnosis, Chiba University Graduate School of Medicine, Chiba, Japan; 5 Neurology Division, Department of Internal Medicine, Sakura Medical Center, Toho University, Sakura, Japan; Florey Institute of Neuroscience and Mental Health, The University of Melbourne, AUSTRALIA

## Abstract

**Objective:**

To determine the ability of sphincter electromyography (EMG) and post-void residual urine volume (PVR) during a free-flow study and a pressure-flow study (PFS) for distinguishing multiple system atrophy (MSA) from Parkinson’s disease (PD).

**Methods:**

We retrospectively reviewed 241 case records; both urodynamic study and sphincter EMG were performed in patients with MSA (*n* = 147) and PD (*n* = 94).

**Results:**

There was a statistically significant difference (*p* < 0.01) in the mean PVR during the free-flow study (113.1 ± 7.5 mL in MSA and 40.4 ± 3.8 mL in PD), mean PVR during PFS (230.1 ± 12.6 mL in MSA and 71.7 ± 6.6 mL in PD), and mean duration of MUP for sphincter EMG (9.3 ± 0.1 ms in MSA and 7.7 ± 0.1 ms in PD). The area under the curve used for differentiating MSA from PD was 0.79 and 0.73 for PVR during PFS and the free-flow study, respectively. There was a mean duration of 0.69 ms for the sphincter EMG.

**Conclusions:**

The present results suggested that PVR was more appropriate than sphincter EMG for differentiating MSA from PD.

## Introduction

Although it is often difficult to differentiate multiple system atrophy (MSA) from Parkinson’s disease (PD), examining urinary dysfunction might be helpful for diagnosing MSA in cases with more severe urinary voiding dysfunction than in PD [1–3]. We previously reported that the post-void residual urine volume (PVR) and sphincter electromyography (EMG) might be helpful for differentiating MSA from PD [1–4]. Urinary voiding dysfunctions are examined by a urinary symptom questionnaire and measuring the PVR in clinical practice. Bladder contractility can be evaluated by performing a pressure-flow study (PFS) [5]. Neurogenic changes in the anal sphincter muscle can be evaluated by performing an external anal sphincter (EAS)-EMG [6].

Although the utility of a sphincter EMG for differentiating MSA from PD has been controversial [7,8], many studies revealed that the prevalence of neurogenic changes was higher in MSA than that in PD [9–11]. Moreover, multiple studies (including our previous studies) suggested that the PVR of patients with MSA was significantly larger than that of patients with PD [1,3,12]. However, the optimal parameters for differentiating MSA from PD remain unknown. Furthermore, some patients with MSA exhibited significantly larger PVR during PFS than those during the free-flow study. We aimed to determine the ability of sphincter EMG and PVR (both during the free-flow study and PFS) for distinguishing MSA from PD using a receiver operating characteristic (ROC) analysis. We also aimed to determine the PVR cutoff points (during the free-flow study and PFS) and the mean duration of MUPs using the Youden index [13].

## Methods

### Standard protocol approval, registration, and patient consent

The Chiba University institutional review committees and an ethical standard committee approved this study. We obtained oral informed consent from all patients, which was recorded in their medical notes.

### Patient characteristics

We retrospectively reviewed 241 case records; both a urodynamic study and a sphincter EMG were performed for patients with MSA (*n* = 147; 91 men and 56 women, mean age 64.1 ± 0.53 years, mean duration 3.2 years) and PD (*n* = 94; 63 men and 31 women, mean age 66.2 ± 0.46 years, mean duration 3.2 years). All patients who experienced any lower urinary tract symptoms were assessed by a detailed urinary symptom questionnaire [1,3]. Patients with MSA were diagnosed with probable or possible MSA based on the Gilman et al [14] second consensus criteria. The majority of the patients were examined by brain magnetic resonance imaging (MRI). Patients with PD were diagnosed based on the United Kingdom Parkinson’s Disease Society Brain Bank clinical diagnostic criteria [15]. Radiological examinations were performed by obtaining a brain MRI or computed tomography scan.

We excluded patients with comorbid conditions, such as diabetic neuropathy, lumbar spondylosis, or benign prostatic hyperplasia, and with a history of pelvic organ surgery, which might collectively affect the results of PVR and sphincter EMG.

### Urodynamic study

A urodynamic study was performed by a urologist and neurologists who were familiar with urodynamic study findings and sphincter EMG. The neurologists in this study examined the lower urinary tract dysfunction associated with PD and MSA.

PVR during the free-flow study (normal volume, <50 mL) was measured using transurethral catheterization after voiding. Cystometry was performed using a urodynamic computer (Janus; Life-Tec Inc., Houston, TX, USA). The sphincter EMG was performed using an EMG computer (Neuropack Sigma; Nihon Kohden Inc., Tokyo, Japan). The EMG of the anal sphincter was recorded by inserting a coaxial needle electrode into the EAS muscles continuously during the measurement collection. An 8-Fr double-lumen catheter was inserted transurethrally, and water (saline) cystometry was performed at an infusion rate of 50 mL/min in the sitting position. The rectal pressure was measured using a balloon catheter, which was simultaneously electronically subtracted from the intravesical pressure. We performed PFS after the cystometry. Both the free-flow study and the PFS were performed in the sitting position. PVR was measured after evacuation during the free-flow study (PVR during free-flow study) and PFS (PVR during PFS). Tracings of water cystometry, anal sphincter EMG, and PFS were reviewed by the neurologists and urologist who are familiar with urodynamic studies. Falsely high *Q*max values during abdominal straining were excluded.

Abnormal urodynamic findings during the storage phase are as follows: (1) detrusor overactivity was defined as involuntary detrusor contractions during the filling phase; (2) impaired bladder contractility was classified as abnormal urodynamic findings during the voiding phase; (3) the degree of detrusor contraction was examined using Schäfer’s nomogram (classifying detrusor contractility as strong, normal, weak, and very weak depending on detrusor pressure and flow rate); and (4) the methods, definitions, and units employed conformed to the standards recommended by the International Continence Society [5].

### EAS-EMG

A concentric needle electrode (needle diameter, 0.46 mm; Alpine Biomed, Skovlunde, Denmark) was inserted into the most superficial layer of the anal sphincter muscle under audio guidance. The examiner inserted the needle into the right (5 o’clock position) and left (7 o’clock position) sphincter muscles and performed the MUP analyses separately. Five MUPs were stored on each side. The examiner manually adjusted the position of the needle electrode until continuous firing activities of 3–5 MUPs were obtained visually. The rise time of the MUP was 300–500 μs. The range of sites from which the MUPs were recorded was approximately 1 cm from the anal orifice to a depth of 3–6 mm. A gain of 100 μV at 5 ms/div was used. The amplifier filter was set at 5–10 kHz.

MUPs were stored in the computer only when the amplitude reached the threshold determined by the examiner. The examiner manually adjusted the threshold by moving the cursor to detect the visually confirmed 3–5 MUPs; subsequently, a total of 64 MUPs was stored in the computer. The four most similar MUPs among the 64 stored MUPs were identified using the auto MUP analysis software equipped with the EMG computer. The stored 64 MUPs were numbered as MUP1, MUP2, … and MUP64. The MUP1 was initially registered as a template wave via the MUP analysis software, and the correlational coefficient between MUP1 and MUP2 was calculated. If the correlation coefficient exceeded 0.94, the software regarded MUP2 to be equivalent to MUP1. Otherwise, MUP2 was considered to be a different wave from MUP1. The software repeated this procedure for the other MUPs, and the four most similar MUPs were finally identified. The onset of MUP was automatically determined by the software when both the slope and the voltage exceeded the predefined thresholds (onset slope, 5 μV/ms; onset level, 20 μV) above or below the baseline voltage in this study. The same procedure was used to determine the termination. The duration was automatically determined consequently. The examiner reviewed the wave form and manually set the cursor to include late components. Late components were defined as the highly stable amplitude separated from the initial part of the complex by an isoelectric period of several milliseconds [11]. The above procedures were repeated to obtain 10 different MUPs by moving the position of the electrode. The mean duration of the 10 different MUPs was obtained. The neurogenic change was diagnosed when the mean duration of the MUPs (including late components) was >10 ms. The method for performing an EAS-EMG and the diagnostic criteria of a neurogenic change in the EAS-EMG was congruent with those used in a previous study [2]. All EMG recordings were performed at the Chiba University, and all the results in this study were examined using the same criteria. All our authors had an experience of working at the Chiba University; therefore, they understood our duration criteria very well.

### Statistical analysis

SPSS version 22.0 (IBM, Armonk, NY, USA) software was used for statistical analysis. All data are expressed as the mean ± SEM. A Mann–Whitney *U*-test was performed to compare the differences in the PVR (during free-flow study and PFS) and the mean duration of the MUPs between MSA and PD. We performed an ROC analysis to determine the best parameter (PVR [during free-flow study and PFS] or mean duration of the MUPs in the EAS-EMG) to differentiate between MSA and PD. The value that maximized the difference between the sensitivity and the false-positive rate (Youden index) was selected as the cutoff value.

## Results

All patients with PD exhibited a clear and dramatic beneficial response to dopaminergic therapy. Radiological findings were normal in patients with PD. Many patients with MSA showed orthostatic hypotension, which satisfied the probable or possible criteria of MSA [16]. None of the patients with MSA exhibited a significant beneficial response to dopaminergic therapy. Atrophy of the putamen was found in the parkinsonian phenotype of MSA, whereas atrophy of the pons, middle cerebellar peduncle, or cerebellum was found in the cerebellar phenotype of MSA. One patient with PD and three patients with MSA were taking anticholinergic drugs, four patients with MSA were taking alpha-blockers, and one patient with PD and one patient with MSA were taking a beta 3 adrenoceptor agonist.

### PVR, mean duration of MUP in sphincter EMG, and bladder contractility

**Fig 1:**
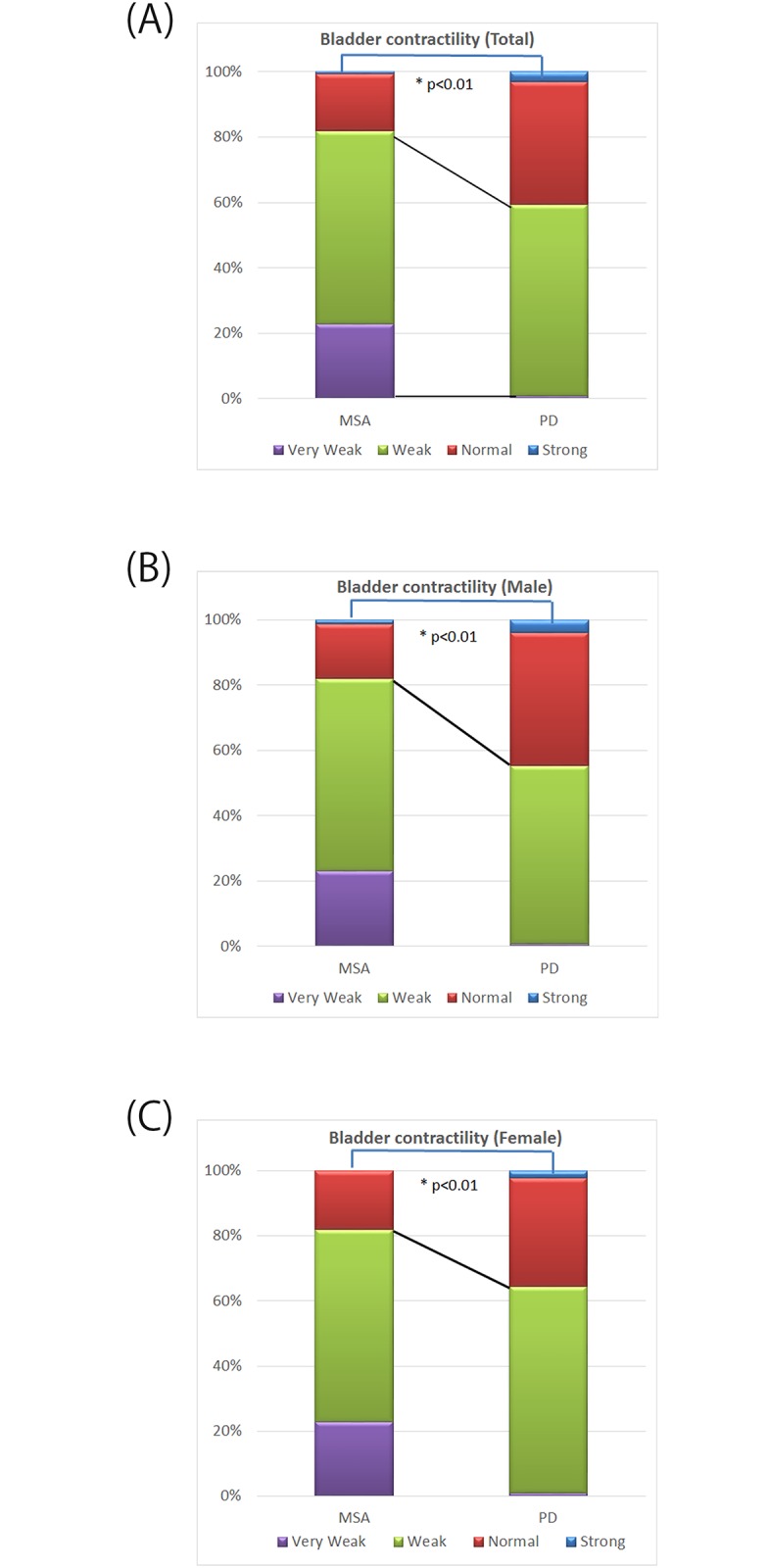
Bladder contractility as evaluated by Schäfer’s nomogram. Bladder contractility was significantly impaired in multiple system atrophy compared with that in Parkinson’s disease for all patients (A), men (B), and women (C).

Statistical significance (*p* < 0.01) was observed for the mean PVR during the free-flow study (116.2 ± 8.7 mL in MSA and 45.6 ± 7.3 mL in PD), the mean PVR during PFS (238.4 ± 14.9 mL in MSA and 78.2 ± 11.3 mL in PD), and the mean duration of MUP in sphincter EMG (9.30 ± 0.18 ms in MSA and 7.77 ± 0.18 ms in PD) in both male and female patients. Bladder contractility evaluated by Schäfer’s nomogram was significantly impaired in patients with MSA compared with that in patients with PD, owing to the higher prevalence of weak and very weak detrusor contractility in patients with MSA (Fig 1A).

In patients with PD, the mean PVR during the free-flow study was 52.1 ± 10.4 mL in men and 32.5 ± 6.5 mL in women (*p* = 0.11). The mean PVR during PFS was 86.4 ± 14.8 mL in men and 60.8 ± 17.2 mL in women (*p* = 0.29). The mean duration of MUPs via the sphincter EMG was 7.8 ± 0.3 ms in men and 7.7 ± 0.2 ms in women (*p* = 0.99; Table 1).

**Table 1:** PVR during the free-flow study and PFS and the mean duration of MUPs in patients with PD.

		Mean ± SEM	*p*-value
PVR (mL) during the free-flow study	Male	52.15 ± 10.48	*p* = 0.11
	Female	32.54± 6.57	
PVR (mL) during the PFS	Male	86.47± 14.87	*p* = 0.29
	Female	60.83± 17.22	
Mean duration (ms) of MUP	Male	7.81± 0.34	*p* = 0.90
	Female	7.76 ± 0.22	

MUP, motor unit potential; MSA, multiple system atrophy; PD, Parkinson’s disease; PVR, post-void residual; PFS, pressure-flow study

In patients with MSA, the mean PVR during the free-flow study was 122.4 ± 11.4 mL in men and 112.0 ± 14.8 mL in women (*p* = 0.58). The mean PVR during PFS was 257.0 ± 19.5 mL in men and 200.0 ± 24.0 mL in women (*p* = 0.07). The mean duration of MUP for the sphincter EMG was 9.3 ± 0.2 ms in men and 9.1 ± 0.3 ms in women (*p* = 0.61; Table 2).

**Table 2:** PVR during the free-flow study and PFS and the mean duration of MUPs in patients with MSA.

		Mean ± SEM	*p*-value
PVR (mL) during the free-flow study	Male	122.4 ± 11.46	*p* = 0.58
	Female	112.0 ± 14.88	
PVR (mL) during the PFS	Male	257.0 ± 19.5	*p* = 0.07
	Female	200.0 ± 24.03	
Mean duration (ms) of MUP	Male	9.35 ± 0.23	*p* = 0.61
	Female	9.14 ± 0.32	

MUP, motor unit potential; MSA, multiple system atrophy; PD, Parkinson’s disease; PVR, post-void residual; PFS, pressure-flow study

The mean PVR and duration of MUP for the sphincter EMG were significantly larger and longer, respectively, in patients with MSA than in patients with PD of both sexes (*p* < 0.001).

Bladder contractility evaluated by Schäfer’s nomogram was significantly impaired in patients with MSA compared with that in patients with PD, owing to the higher prevalence of weak and very weak detrusor contractility in patients with MSA of both sexes (Fig 1B and 1C).

### ROC analysis

**Fig 2:**
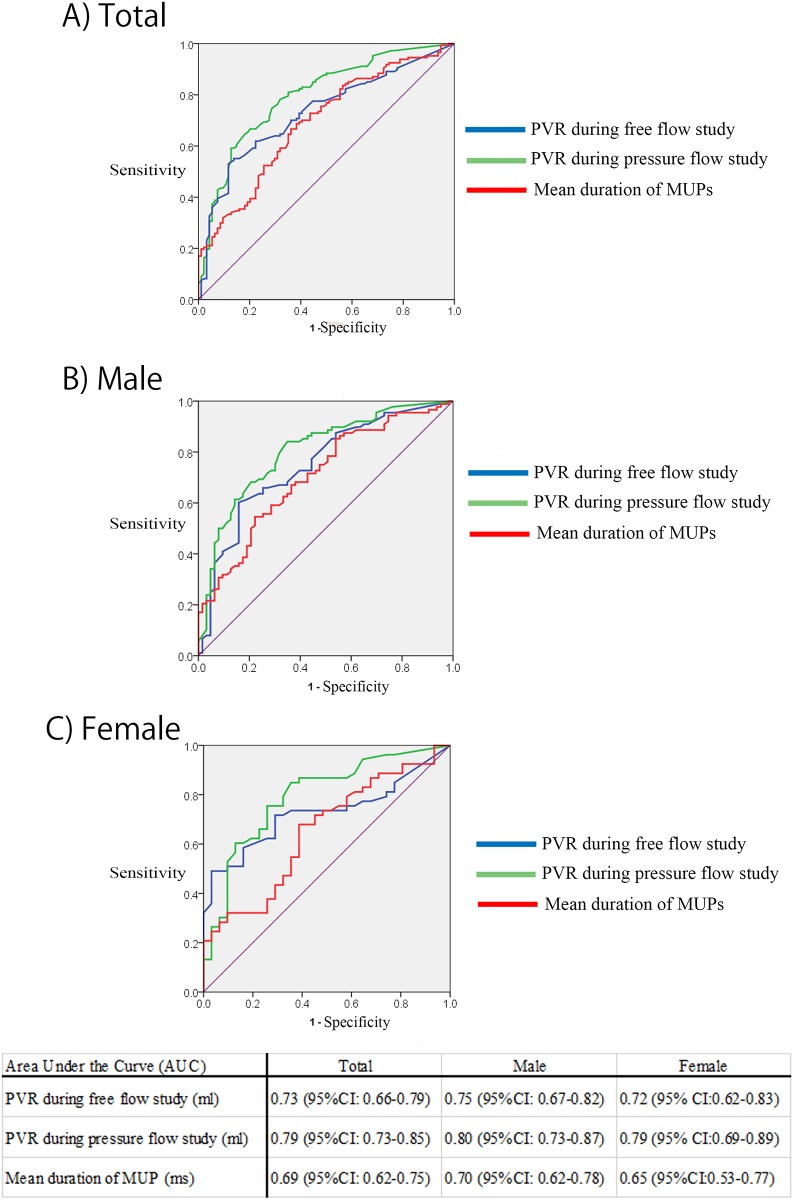
Receiver operating characteristic analysis of post-void residual (PVR) urine volume during the free-flow and pressure-flow studies and the mean duration of motor unit potentials (MUPs) in distinguishing MSA from PD. The area under the curve (AUC) of the PVR (during free-flow and pressure-flow studies) and the mean duration of MUPs are depicted for all patients (A), men (B), and women (C). AUC values are depicted below.

The area under the curve (AUC) was used to differentiate MSA from PD was 0.79 (95% confidence interval (CI): 0.73–0.85) for PVR during PFS, 0.73 (95% CI: 0.66–0.79) for PVR during the free-flow study, and 0.69 (95% CI: 0.62–0.75) for the mean duration of MUPs. The AUC for PVR during PFS and the free-flow study was significantly larger than that for the mean duration of the sphincter EMG (*p* < 0.01; Fig 2A).

In men, the AUC used to differentiate MSA from PD was 0.80 (95% CI: 0.73–0.87) for PVR during PFS, 0.74 (95% CI: 0.67–0.82) for PVR during the free-flow study, and 0.70 (95% CI: 0.62–0.78) for the mean duration of the sphincter EMG. The AUC for PVR during PFS and the free-flow study was significantly larger than that for the mean duration of the sphincter EMG (*p* < 0.01; Fig 2B).

In women, the AUC used to differentiate MSA from PD was 0.79 (95% CI: 0.69–0.89) in PVR during PFS, 0.72 (95% CI: 0.62–0.83) for PVR during the free-flow study, and 0.65 (95% CI: 0.53–0.77) for the mean duration of the sphincter EMG. The AUC for the PVR during PFS and the free-flow study was significantly larger than that for a mean duration of the sphincter EMG (*p* < 0.01; Fig 2C).

### Cutoff values, sensitivity, and specificity of the PVR and mean duration of MUP

The cutoff values calculated from the Youden index for all patients (both sexes) were 87 mL for the PVR during the free-flow study, 85 mL for the PVR during PFS, and 7.9 ms for the mean duration of MUPs (Table 3). Sensitivity for all patients (both sexes) was 0.53 for the PVR during the free-flow study, 0.71 for the PVR during PFS, and 0.67 for the mean duration of the MUPs. Specificity for all patients (both sexes) was 0.88 for the PVR during the free-flow study, 0.71 for the PVR during PFS, and 0.54 for the mean duration of the MUPs (Table 4).

**Table 3:** Cutoff values calculated from the Youden index.

	Cutoff value		
	Total	Male	Female
PVR during the free-flow study (mL)	87	85	95
PVR during the PFS (mL)	85	85	72
Mean duration of MUP (ms)	7.9	8.9	8.3

MUP, motor unit potential; PFS, pressure-flow study; PVR, post-void residual.

**Table 4:** Sensitivity and specificity of the cutoff values.

	Sensitivity			Specificity		
	Total	Male	Female	Total	Male	Female
PVR during the free-flow study (mL)	0.53	0.6	0.49	0.88	0.84	0.96
PVR during the PFS (mL)	0.71	0.84	0.75	0.71	0.61	0.74
Mean duration of MUP (ms)	0.67	0.54	0.67	0.54	0.77	0.61

MUP, motor unit potential; PFS, pressure-flow study; PVR, post-void residual.

## Discussion

Our results suggest that the PVR was more appropriate than the mean duration of the MUPs in a sphincter EMG for differentiating MSA from PD. In particular, the AUC for PVR during PFS was larger than that during the free-flow study. Sex differences were not statistically significant in this study. Although we previously reported several papers regarding the lower urinary tract dysfunction of PD and MSA using a urodynamic study and an anal sphincter EMG [1–3], the present study advanced the direct statistical comparison between the PVR and the anal sphincter EMG, which has not been previously reported.

It is difficult to differentiate MSA from PD, at least during the early stages because some patients with MSA (particularly the parkinsonian phenotype) respond to antiparkinsonian drug treatments [16], and many neurologists are not familiar with the evaluation of urinary dysfunction. Although most neurologists might check the presence or absence of urinary symptoms (e.g., urinary incontinence or incomplete bladder emptying) in patients presenting with parkinsonism [16 17], parkinsonism cannot be diagnosed by such urinary symptoms alone. Urinary dysfunction is one of the non-motor symptoms associated with PD [18,19] and the Movement Disorder Society-sponsored revision of the Unified Parkinson’s Disease Rating Scale includes a questionnaire relating to urinary symptoms in Part 1 [20]. It is important to understand the differences in the severity and pattern of urinary dysfunction between PD and MSA. As we previously reported, urinary dysfunction in PD is characterized by urinary storage dysfunction (e.g., urinary urgency or urgent urinary incontinence) with mild urinary voiding dysfunction [19, 21], whereas patients with MSA exhibit more severe urinary storage and voiding dysfunction [1–3]. Although it is common for many patients with MSA to require clean intermittent catheterization and eventually an indwelling urinary catheter [12], patients with PD rarely require either of these. The remarkable difference between PD and MSA regarding urinary dysfunction is the severity of the urinary voiding dysfunction [3]. The most reliable examination in patients with a voiding dysfunction is measuring the PVR. It might be helpful for neurologists to measure the PVR to distinguish MSA from PD. It is also important to exclude comorbid conditions (e.g., diabetic neuropathy, lumbar spondylosis, and benign prostatic hyperplasia), as well as a history of pelvic organ surgery, which collectively might lead to a large PVR.

PVR usually differs between the sexes owing to anatomical differences. Although the present study did not show any significant sex differences in the PVR, men tended to have a larger PVR. However, attention must be paid to sex differences while examining urinary voiding dysfunctions [1].

It is interesting that PVR during PFS was superior to the PVR during the free-flow study for distinguishing MSA from PD. Although the PVR during PFS is less physiologically relevant compared with the PVR during the free-flow study, we showed that the PVR during PFS might have the potential to be used as a diagnostic tool in differentiating MSA from PD. Slightly increasing the urethral resistance by inserting a narrow urethral catheter (8 Fr in this study) may have a significant influence on the voiding function in patients with impaired bladder contractility, leading to a markedly larger PVR during PFS. In the present study, we revealed that bladder contractility as evaluated by the Schäfer nomogram was significantly impaired in patients with MSA of both sexes compared with patients with PD; therefore, PVR during PFS was superior to PVR during a free-flow study in distinguishing MSA from PD.

Since the EAS is innervated by Onuf’s nucleus, which is known to be degenerated in MSA [4], an EAS-EMG has been performed for the diagnosis of MSA [7–11]. Moreover, we revealed that the mean duration of MUPs had a higher AUC than polyphasicity and amplitude of MUPs by performing an ROC analysis in our previous study [2]; thus, we used only the mean duration of MUPs among the parameters of MUPs. We showed that PVR was superior to mean duration of MUPs in the sphincter EMG for distinguishing MSA from PD.

Although we found that the PVR during a PFS was superior for distinguishing MSA from PD, measuring the PVR during a free-flow study may be more appropriate during routine examinations, than performing a PFS or sphincter EMG, which are invasive and more difficult to perform.

It is also important to discuss the cutoff value for PVR during the free-flow study. A cutoff value of 100 mL in the free-flow study has been empirically and widely used for the differentiation of MSA in previous studies and the diagnostic algorithm for urological disorders. A cutoff value of 87 mL for all patients (both sexes) was calculated with relatively high specificity (0.88) from the Youden index in this study. An empirically used cutoff value of 100 mL during the free-flow study might be highly specific for distinguishing MSA from PD. However, the sensitivity of PVR during the free-flow study was 0.53, which was not high, and may be lower than the sensitivity of the mean duration of MUP in the anal sphincter EMG. An ROC analysis calculates the sensitivity and specificity of a test for every possible cutoff point. The AUC of the ROC curve may be used to assess the diagnostic accuracy of a test and compare the usefulness of a different test [22, 23]. The utility of diagnostic tests cannot be simply determined by the sensitivity in the ROC analysis. Since the AUC of PVR during the free-flow study was significantly larger than that of the mean duration of MUP in EMG, an ROC analysis might suggest that the PVR during the free flow study was more appropriate than the anal sphincter EMG as a diagnostic test in this study.

It should be noted that the relationship between the present study and our previous studies [1–3, 12, 24] examining the diagnostic utility of PVR and anal sphincter EMG in differentiating PD and MSA. We have previously reported that PVR [12] and anal sphincter EMG [2, 6, 24] are useful for differentiating MSA from PD, separately. However, the aim of the present study was to perform a direct comparison of the PVR and anal sphincter EMG as a diagnostic utility for differentiating MSA from PD, which have not been previously examined. The aim of the present study and our previous studies [2, 6, 12, 24] was different. As we have previously reported, time-dependent changes in the PVR [12] and the mean duration of MUP in anal sphincter EMG [24] are also important. However, we did not perform repeated measures over time or changes in the disease duration for all patients included in the present study.

There are some limitations to this study. First, because the diagnosis of PD and MSA was based on clinical criteria, a detailed urodynamic study may not contribute to clinical practice. However, the PVR during the free-flow study had relatively high specificity. The present study revealed that an empirically used cutoff value of 100 mL might be highly specific. Second, the present study did not include any autopsy data. Third, the present study lacked healthy controls. However, it is unrealistic to perform an invasive urodynamic study and EAS-EMG in age-matched healthy controls.

It is also relevant to include the disease duration in describing the cohort and to control for this variable in the ROC analysis. However, since our cohort includes almost exclusively patients in early disease stages, including disease duration would not usefully add to the ROC analysis.

It is also important to mention that a portion of the patient cohort in this study overlapped with our previous study. However, we excluded any patients with comorbid conditions in this study. Moreover, we excluded any patients who could not void at all during PFS, in which case the PVR during PFS is not available. Since our previous study did not examine the PVR during PFS, the number of patients with PD and MSA is different between the present study and our previous study. Although some of the patients included in this study overlapped with our previous study, this study used different statistical analyses and provides different clinical utility.

## Conclusion

Our findings suggest that the PVR was more appropriate than the mean duration of sphincter EMG for differentiating MSA from PD. In particular, the AUC for PVR during PFS was larger than that during the free-flow study.
